# Reducing Health Risks from Indoor Exposures in Rapidly Developing Urban China

**DOI:** 10.1289/ehp.1205983

**Published:** 2013-05-10

**Authors:** Yinping Zhang, Jinhan Mo, Charles J. Weschler

**Affiliations:** 1 Department of Building Science, School of Architecture, Tsinghua University, Beijing, China; 2 Environmental and Occupational Health Sciences Institute, University of Medicine and Dentistry of New Jersey (UMDNJ)–Robert Wood Johnson Medical School and Rutgers University, Piscataway, New Jersey, USA; 3 International Centre for Indoor Environment and Energy, Technical University of Denmark, Lyngby, Denmark

**Keywords:** air pollutants, birth defects, cancer, endocrine disruptors, indoor air quality, urbanization

## Abstract

Background: Over the past two decades there has been a large migration of China’s population from rural to urban regions. At the same time, residences in cities have changed in character from single-story or low-rise buildings to high-rise structures constructed and furnished with many synthetic materials. As a consequence, indoor exposures (to pollutants with outdoor and indoor sources) have changed significantly.

Objectives: We briefly discuss the inferred impact that urbanization and modernization have had on indoor exposures and public health in China. We argue that growing adverse health costs associated with these changes are not inevitable, and we present steps that could be taken to reduce indoor exposures to harmful pollutants.

Discussion: As documented by China’s Ministry of Health, there have been significant increases in morbidity and mortality among urban residents over the past 20 years. Evidence suggests that the population’s exposure to air pollutants has contributed to increases in lung cancer, cardiovascular disease, pulmonary disease, and birth defects. Whether a pollutant has an outdoor or an indoor source, most exposure to the pollutant occurs indoors. Going forward, indoor exposures can be reduced by limiting the ingress of outdoor pollutants (while providing adequate ventilation with clean air), minimizing indoor sources of pollutants, updating government policies related to indoor pollution, and addressing indoor air quality during a building’s initial design.

Conclusions: Taking the suggested steps could lead to significant reductions in morbidity and mortality, greatly reducing the societal costs associated with pollutant derived ill health.

Rapid industrial and economic development in China over the past three decades has resulted in a large migration from rural areas to cities, urban growth, and modernization and a concomitant increase in urban air pollution. As shown in Figure 1, during the period from 1990 to 2010 the urban population more than doubled, net urban residential building area grew from 4 billion to 21 billion m^2^, and the number of motor vehicles increased from 5 million to 78 million. The nature of indoor environments also changed as a consequence of using the different building materials, construction practices, and climate control that accompanied the replacement of low-rise dwellings with high-rise apartment buildings. Emblematic of changing building materials is the increased production of synthetic wood [from 15 million m^3^ in 1999 to 154 million m^3^ in 2010 (State Forestry Administration–People’s Republic of China 2011)], and indicative of changing climate control is the increased presence of mechanical cooling in urban residences [from < 1 million air conditioners in 1990 to > 100 million in 2010 (National Bureau of Statistics–People’s Republic of China 2011)]. As a result of these and other developments, China’s cities have been experiencing rapid and dramatic changes in outdoor and indoor environments. These changes have affected hundreds of millions of people.

**Figure 1:**
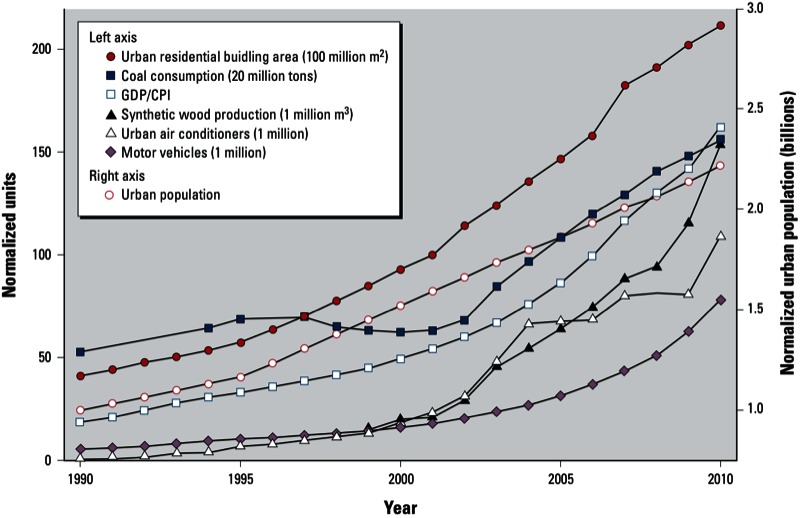
Various indicators of rapid modernization in China during the period 1990–2010. GDP units: billion Chinese yuan; CPI in 1990 = 100; urban population normalized by 1990 value (301.95 million). Abbreviations: CPI, consumer price index; GDP, gross domestic product. Data for synthetic wood production from State Forestry Administration–People’s Republic of China (2011). All other data from National Bureau of Statistics–People’s Republic of China (2011).

Here we discuss how urbanization and modernization in China have resulted in changes in indoor exposures to pollutants that originate both indoors and outdoors. We then examine ill health associated with these pollutant exposures and suggest policies that might be implemented to mitigate these adverse health effects.

## Discussion

*The changing nature of indoor exposure to indoor and outdoor pollutants*. The inhabitants of China’s cities spend most of their time indoors (Wang et al. 2008). Their indoor environments contain pollutants from outdoor sources (e.g., coal and oil combustion used in power plants, industry, and heating; motor vehicles; fugitive emissions; metallurgy; and biomass burning) as well as indoor sources (e.g., occupants, unvented combustion, building materials, furnishings, paint, floor and wall coverings, cleaning products, pesticides, appliances, and electronics). Pollutants emitted by indoor sources have changed dramatically over the past 30 years, reflecting the increased use of plastics, polymeric floor and wall coverings, synthetic wood products, and synthetic cleaning agents (Wang et al. 2010; Weschler 2009). These changes have led to higher concentrations of various organic compounds in the indoor environments of China’s cities [Bai et al. 2002; Edward et al. 2003; Guo and Kannan 2011; Hsu et al. 2012; Liu ZR et al. 2012; Wang et al. 2010; Wu et al. 2003; Zhang et al. 2009; see also Supplemental Material, pp. 2–3 and Table S1 (http://dx.doi.org/10.1289/ehp.1205983)]. The increased use of mechanical cooling (air conditioning) has decreased ventilation rates during warm periods and amplified exposures to pollutants from indoor sources (Meng et al. 2009).

Outdoor air in China tends to be more polluted in cities than in rural and semi-rural areas, reflecting emissions from power plants, industrial facilities, and motor vehicles. Urban levels of PM_10_, PM_2.5_ (particles with aerodynamic diameters of ≤ 10 or ≤ 2.5 µm, respectively), ozone (O_3_), nitrogen oxides, and sulfur dioxide (SO_2_) are among the highest in the world (Kan et al. 2012; Zhang et al. 2012). In Beijing in 2011, the average annual level of PM_2.5_ was roughly an order of magnitude higher than that in Boston, Massachusetts; Chicago, Illinois; or Washington, DC (Dominici and Mittleman 2012). These outdoor pollutants are transported indoors via ventilation and infiltration. Given the amount of time that people spend indoors, for many urban residents the major fraction of their exposure to “outdoor pollutants” occurs indoors (Chen C et al. 2012a, 2012b; Chen and Zhao 2011; Hodas et al. 2012; Meng et al. 2009; Mullen et al. 2011; Wang et al. 2008).

**Figure 2:**
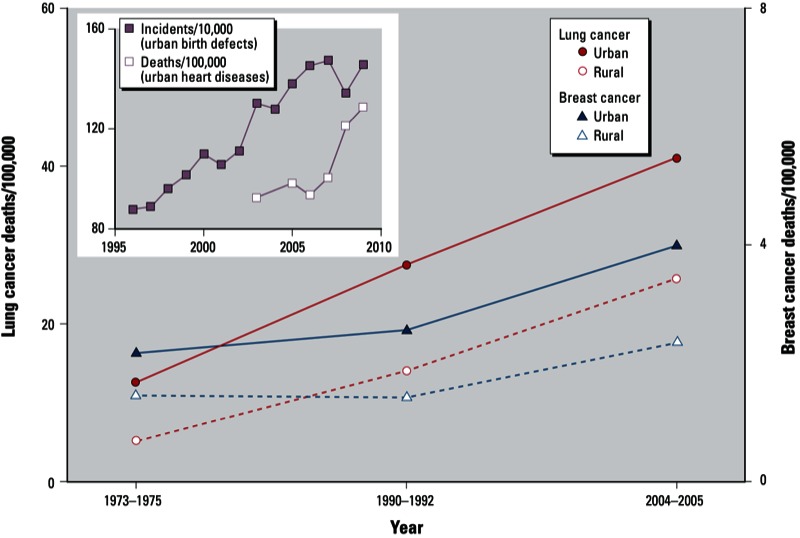
Increasing mortality or incidence rates for different health end points in China. Lung cancer deaths include those attributable to smoking. Excluding deaths attributable to smoking (Gu et al. 2009), the 2004–2005 rates were 16.5 (urban) and 8.2 (rural) per 100,000 persons. Data for lung cancer, breast cancer, and heart disease from Ministry of Health–People’s Republic of China (2010). Data for birth defects from Ministry of Health–People’s Republic of China (2011).

*Health consequences*. China has experienced significant increases in certain diseases that have been linked to air pollution [see Supplemental Material, Figure S1 (http://dx.doi.org/10.1289/ehp.1205983)]. These include lung cancer [ranked first among cancer mortalities in urban China (Ministry of Health–People’s Republic of China 2010)], cardiovascular disease, pulmonary disease, and birth defects. Figure 2 illustrates the increase over the last three decades in deaths/100,000 persons for lung cancer in urban and rural regions. Mortality rates for these cancers are higher in urban than rural regions, and the difference between urban and rural mortality rates has been increasing. Smoking is responsible for a majority of lung cancer deaths. For the year 2005, Gu et al. (2009) estimated that 137,900 urban lung cancer deaths (24.5/100,000 persons) and 130,700 rural lung cancer deaths (17.5/100,000 persons) were attributable to smoking. Subtracting these smoking-related lung cancer mortality rates from the total lung cancer mortality rates during this period leaves nonsmoking attributable lung-cancer mortality rates of 16.5/100,000 persons in urban areas and 8.2/100,000 persons in rural areas. Recently, in a prospective study of almost 71,000 subjects residing in 31 cities in China, outdoor air pollution was conclusively associated with lung cancer as well as with cardiopulmonary mortality (Cao et al. 2011). A large fraction of outdoor pollutants are actually inhaled indoors.

Figure 2 also shows mortality rates for breast cancer in China. The U.S. Institute of Medicine (2012) recently concluded that exposure to certain pollutants found in motor vehicle exhaust (e.g., benzene, ethylene oxide, 1,3-butadiene) may be linked to a higher breast cancer risk. Furthermore, they judged that it was biologically plausible that bisphenol A and nonylphenol, common indoor pollutants (Rudel et al. 2003), contribute to breast cancer, but that further research was necessary to confirm this. In addition to bisphenol A and nonylphenol, urban indoor environments in China contain other chemicals that may function as endocrine disruptors (Guo and Kannan 2011; Wang et al. 2010).

The inset in Figure 2 shows normalized incidents of birth defects from 1996 to 2009 for urban residents of China. During this period, the number of incidents per 10,000 persons in urban areas almost doubled, whereas the increase was much smaller (22%) in rural areas (Ministry of Health–People’s Republic of China 2011).

Li et al. (2012) have discussed these trends, noting that less-polluted western China has a lower incidence of birth defects than the more heavily polluted coastal cities (Ministry of Health–People’s Republic of China 2011); they summarized several studies that made associations between environmental pollutants in China and birth defects. These associations include polychlorinated biphenyls (PCBs) and hypospadias (Dai et al. 2011); polycyclic aromatic hydrocarbons (PAHs) and children’s neurobehavioral development (Perera et al. 2008); and PAHs, *o,p*-dichlorodiphenyltrichloroethane (*o,p*-DDT), and α-hexachlorocyclohexane and neural tube defects (Ren et al. 2011). Such pollutants are common constituents of indoor air and dust (Liu ZR et al. 2012; Wang et al. 2010; Weschler and Nazaroff 2008). Zhang et al. (2009) found that *in utero* exposures to two common indoor pollutants, di(*n*-butyl) phthalate (DnBP) and di(2-ethylhexyl) phthalate (DEHP), were associated with low birth weight in a dose-dependent manner.

The inset in Figure 2 also shows that deaths from heart disease for urban residents have increased from 94/100,000 persons in 2003 to 129/100,000 in 2009. Substantial evidence indicates that airborne particles contribute to these increases (Brook et al. 2010). In an assessment of population exposure to airborne particles in Chongqing, China, Wang et al. (2008) concluded that indoor PM_10_ levels within residences were the largest contributor to population-weighted PM_10_ exposure.

Between 2005 and 2009, the urban death rate from pneumonia increased from 6.0 to 12.6/100,000 persons, while the rural rate increased from 7.1 to 9.8/100,000 persons (Ministry of Health–People’s Republic of China 2010). In the United States, hospital admissions for pneumonia correlate positively with outdoor O_3_ and PM_10_ levels (Medina-Ramón et al. 2006). In the cities of Kaohsiung and Taipei, various outdoor air pollutants have been associated with hospitalization for pneumonia (Cheng et al. 2007; Chiu et al. 2009). Indoors, cooking, smoking, and unvented combustion further contribute pollutants associated with pneumonia.

Before, during, and after the Beijing Olympics, biomarkers of inflammation and thrombosis related to cardiovascular disease were measured in a group of healthy young medical students (Rich et al. 2012). When air pollution decreased during the Olympics, several biomarkers related to platelet adhesion and activation improved significantly. After the Olympics, when air pollution approached pre-Olympic levels, these biomarkers reverted to pre-Olympic levels. As noted by the investigators, the subjects’ exposure to air pollution occurred primarily indoors.

The prevalence of asthma among urban Chinese < 14 years of age rose > 50% between 1990 and 2000, reaching 2.0% (Chen YZ 2004). In a 2008 cross-sectional survey of this same age group, the prevalence of asthma in Beijing, Chongqing, and Guangzhou was 3.2%, 7.5%, and 2.1%, respectively; these values were significantly higher than those measured 10 years earlier using the same methodology (Zhao et al. 2010). Some of this increase has been ascribed to outdoor air pollution (Watts 2006). Indoor exposures to certain plasticizers, flame retardants, and pesticides may also be contributing to the increase (Bornehag and Nanberg 2010; Hsu et al. 2012; Wang et al. 2010).

Increases in premature deaths among high-risk groups have been associated with increases in the concentration of outdoor PM_10_ in 16 cities throughout China (Chen RJ et al. 2012); females, the elderly, and those with little education appeared to be more vulnerable. Changes in short-term mortality have also been associated with changes in the levels of O_3_ and nitrogen dioxide (NO_2_) in four cities located in the Pearl River Delta of southern China (Tao et al. 2012). Kan et al. (2012) summarized more than a dozen other epidemiological studies that have found associations between short-term morbidity or mortality and PM_10_, PM_2.5_, O_3_, NO_2_, and SO_2_ levels in various Chinese cities. Recently, average air exchange rates for buildings in different U.S. cities have been shown to partially explain city-to-city differences in mortality associated with O_3_ and PM_10_ (Chen C et al. 2012a, 2012b): the larger the indoor exposure to “outdoor” O_3_ (or PM_10_), the larger the O_3_ (or PM_10_) mortality coefficient.

Epidemiological investigations using outdoor concentrations measured at central monitoring sites are starting points for the evaluation of health effects stemming from indoor exposures to pollutants with outdoor sources. Comparable studies are not feasible for pollutants with indoor sources. Loh et al. (2007) used concentrations of various organic pollutants measured in different indoor environments and outdoors, coupled with a personal exposure model, to evaluate cancer risks from various air pollutants in the United States. They found that the air pollutants most responsible for cancer risk were 1,3-butadiene, formaldehyde, and benzene (using risk factors from the Office of Environmental Health and Hazard Assessment, California Environmental Protection Agency 2005), and that 69% of the total risk came from exposures occurring indoors. Indoor sources contributed 70% of the formaldehyde risk and 20% of the benzene risk. Logue et al. (2012) examined the chronic health impact of indoor air pollutants in the context of disability-adjusted life years (DALYs) lost as a consequence of various adverse health effects. The pollutants whose inhalation was most responsible for the DALY losses were PM_2.5_, acrolein, formaldehyde, and O_3_; the first three typically have strong indoor sources. In the United States, cumulative impacts from the indoor inhalation of pollutants were estimated to be 400–1,100 DALYs lost annually per 100,000 persons. In China, where the indoor concentrations of PM_2.5_, formaldehyde, and many other organic compounds tend to be higher than in the United States [see Supplemental Material, p. 3 and Table S1 (http://dx.doi.org/10.1289/ehp.1205983)], one would anticipate an even greater negative impact.

*Health costs*. The World Bank together with China’s State Environmental Protection Administration have estimated the health costs of outdoor air pollution in China for the year 2003 (World Bank and State Environmental Protection Administration 2007). Only the urban population was used to calculate these costs because they assumed that this was the primary group exposed to outdoor air pollution. If premature deaths are monetized using the “present value” of per capita gross domestic product (GDP) over the remainder of a person’s lifetime, then the economic burden of premature mortality (111 billion yuan; 17 billion U.S. dollars) and morbidity (46.4 billion yuan; 7.3 billion U.S. dollars) added up to 1.2% of China’s GDP. If premature death is monetized using people’s willingness to pay to avoid mortality risks, then the economic burden of premature mortality (394 billion yuan; 62 billion U.S. dollars) and morbidity (126 billion yuan; 20 billion U.S. dollars) added up to 3.8% of China’s GDP. These are conservative estimates based only on the health effects of PM_10_; pollutants with indoor sources were not included in this analysis.

## Recommendations

In the coming two decades, 350 million people are expected to move to China’s cities from rural areas (Lan 2012). Indoor exposures to air pollutants (and their associated health costs) are also anticipated to increase. However, steps could be taken that would reduce indoor exposures to health-damaging pollutants. Examples are listed in Table 1. Some of these warrant further comment:

**Table 1:** Approaches to reduce indoor exposures to health damaging pollutants.

Goal	Suggested actions
Reduce ingress of outdoor pollutants	In mechanically ventilated buildings, use suitable filters to remove particles from the ventilation air; maintain them properly.
	In cities that routinely experience high O_3_ levels, use charcoal filters or catalytic devices to remove O_3_ from the ventilation air.
	Avoid leaks in the building envelope.
Reduce sources of indoor pollutants	Avoid unvented indoor combustion, including tobacco smoking; use exhaust hoods for cooking.
	Develop various low-emitting indoor building materials and furnishings.
	Educate consumers to choose low-emitting indoor building materials and furnishings, especially for large area sources (e.g., flooring, walls).
	Avoid materials containing known or suspected endocrine disruptors. (Not all plasticizers or flame retardants are endocrine disruptors.)
	Control moisture in buildings to minimize mold and mildew.
	Ensure sufficient ventilation to remove pollutants with indoor sources.
	Consider the use of free-standing filtration units to remove indoor pollutants.
Improve government policies	Update “GB-50325–Indoor Environmental Pollution Control of Civil Building Engineering” [currently addresses only radon, formaldehyde, benzene, ammonia, and total volatile organic compounds (TVOCs)] and “GB/T-18883–Indoor Air Quality Standard” (currently addresses only SO_2_, NO_2_, CO (carbon monoxide), CO_2_ (carbon dioxide), NH_3_ (ammonia), O_3_, formaldehyde, benzene, toluene, xylene, benzo[*a*]pyrene, TVOCs, and PM_10_) to include acetaldehyde, acrolein, 1,3-butadiene, chloroform, naphthalene, dichlorobenzene, PM_2.5_, and other indoor pollutants identified as particularly hazardous (Logue et al. 2012; Loh et al. 2007).
	Establish a standard similar to ASHRAE (American Society of Heating, Refrigerating, and Air Conditioning Engineers) Standard 62.1-2010 that requires removing pollutants from ventilation air in cities where pollutants exceed a given threshold.
	Introduce and enforce standards regarding emissions from building materials, flooring, wall coverings, and furniture.
	Establish a labeling system for building materials and furniture that lists hazardous chemical constituents (Liu WW et al. 2012).
	Balance programs to reduce building energy use with health considerations. Focus on approaches that meet both goals (e.g., heat recovery ventilators, nighttime cooling). When there are conflicts, health should be given priority.
Address indoor air quality during a building’s design	From the design stage, schedule meetings between architects, heating, ventilation, and air conditioning engineers, and interior decorators so that, together, they can plan optimal indoor environments.
	Design indoor environments with low-emitting materials and furnishings.
	Design rooms that are easy to clean; avoid thick carpets, velour type wall coverings, plush upholstery and similar surfaces.
	Design buildings so that condensation on interior surfaces, standing water, and plumbing leaks are less likely to occur. This reduces mold growth.

*In mechanically ventilated buildings, use suitable filters.* Filtration of ventilation air is particularly important in schools, hospitals, nursing homes, and other facilities that house sensitive populations. Efficient particle filters are available that have low resistance to airflow, reducing the energy penalty associated with their use (Stephens et al. 2010).
*In cities that routinely experience high O_3_ levels, use charcoal filters.* This approach is not feasible in naturally ventilated buildings. For such buildings, investigators are examining materials that remove O_3_ from the ventilation air passively (Cros et al. 2012; Kunkel et al. 2010).
*Avoid materials containing known or suspected endocrine disruptors.* Not all plasticizers or flame retardants are endocrine disruptors. This action should focus on compounds for which epidemiological and/or animal studies indicate that endocrine disrupting activity is a concern (Vandenberg et al. 2012).
*Control moisture in buildings to minimize mold and mildew.* Dampness in buildings has been consistently associated with adverse health effects, including cough, wheeze, asthma, headache, and airway infection (Bornehag et al. 2001).
*Consider the use of free-standing filtration units.* Free-standing HEPA filtration units placed in children’s bedrooms were found to reduce PM levels by approximately 50% (Batterman et al. 2012). To be effective, such units should process several volumes of room air per hour.
*Balance programs to reduce building energy use with health considerations.* Less energy use leads to a reduction in outdoor pollution and indoor exposure to these pollutants. However, reducing building energy use should not compromise indoor air quality. Heat recovery ventilators reduce energy use while exhausting pollutants with indoor sources (Kovesi et al. 2009); nighttime cooling provides ventilation while typically introducing less outdoor O_3_ than daytime ventilation (Weschler 2006).

## Conclusions

It will take a long time to reduce outdoor air pollution in China’s major cities. In the interim, we judge that the actions outlined in Table 1 can substantially reduce morbidity and mortality resulting from indoor exposures to pollutants with both outdoor and indoor sources. Costs would be incurred in implementing these mitigation strategies. However, given the large health costs attributed to air pollution, the long-term economic benefits of the proposed interventions are likely to exceed their cost. More important, their implementation would improve the quality of life and health for tomorrow’s residents of urban China.

## Supplemental Material

(553 KB) PDF
