# Supplementary material for: Reducing Health Risks from Indoor Exposures in Rapidly Developing Urban China
Source: Environ Health Perspect. 2013 May 10;121(7):751–5. doi: 10.1289/ehp.1205983 (PMC3701998; doi:10.1289/ehp.1205983)
Supplement: (553 KB) PDF [file ehp.1205983.s001.pdf]

Supplemental Material

Reducing Health Risks from Indoor Exposures in Rapidly Developing Urban China

Yinping Zhang, Jinhan Mo, Charles J. Weschler

Table of Contents for Supplementary Materials   Page

Selected pollutant concentrations in urban residences of China   2

Supplemental Material, Table S1. Indoor residential formaldehyde and BTEX …   4

Supplemental Material, Figure S1. Top ten diseases causing death in urban China   5

References   6

Selected pollutant concentrations in urban residences of China

Formaldehyde and BTEX. Table S1 summarizes indoor residential concentrations of formaldehyde, as well as benzene, ethylbenzene, toluene, and xylene (BTEX) measured in different cities within China. Formaldehyde and benzene are of particular concern since they are known carcinogens (IARC 2012). Synthetic wood (e.g., plywood, fiberboard) used in construction and furniture tends to be the major indoor source of formaldehyde (Salthammer 2012). Wang et al. (2007) found that in Beijing, Shanghai, Guangzhou, and Xi'an, the average indoor/outdoor (I/O) ratio for formaldehyde ranged from 1.6 to 6.4 during the summer and from 2.1 to 11 during the winter. This same study found that acetaldehyde, acetone, 2-butanone, butyraldehyde and benzaldehyde also had average I/O ratios higher than unity, indicative of indoor sources. Indoor sources of BTEX include cooking, smoking and aromatic solvent blends used in different consumer products.

Phthalate esters. A number of studies (Guo and Kannan, 2011; Lin et al., 2009; Lu et al., 2012; Wang et al., 2012; Zhang et al., 2013) have measured mass fractions of phthalate esters in indoor dust (μg/g) from cities in China. Concentrations of phthalate esters in air, airborne particles and surface films are expected to scale with the mass fractions of phthalate esters in indoor dust (Weschler and Nazaroff, 2008; 2010). Di(2-ethylhexyl) phthalate (DEHP), di(isobutyl) phthalate

(DiBP) and di(n-butyl) phthalate (DnBP) are three of the more abundant phthalates found in indoor dust in China.

PBDEs. Chen et al. (2008) measured indoor and outdoor concentrations of polybrominated diphenyl ethers (PBDE) in residences, offices, other indoor workplaces and outdoors in Guangzhou, China. The authors estimate that indoor inhalation and ingestion of dust are important contributors to overall intake of PBDE among the urban residents of Guangzhou.

Supplemental Material, Table S1. Indoor residential concentrations of formaldehyde, benzene, ethylbenzene, toluene and xylene measured in different cities within China (mg/m^3).[a,b]

City	**Formaldehyde** (sample date, size)	**Benzene** (sample date, size)	**Toluene** (sample date, size)	**Ethylbenzene** (sample date, size)	**Xylene** (sample date, size)
Beijing	0.18±0.17 (2002-04, 1207)	0.075±0.135 (2002-04, 373)	0.19±0.46 (2002-04, 373)	-	0.085±0.141 (2002-04, 377)
	0.21±0.15 (2003 winter, 530)				
	0.28±0.21 (2003 summer, 389)				
Shanghai	0.10±0.06 (2002-04, 166)	0.127±0.19 (2002-04, 34)	0.17±0.32 (2002-04, 51)	-	0.21±0.39 (2002-04, 51)
	0.21±0.14 (2003 winter, 182)				
Tianjin	0.13±0.08 (2002-04, 154)	0.15±0.17 (2002-04, 68)	0.085±0.127 (2002-04, 125)	-	0.11±0.21 (2002-04, 125)
	0.27±0.17 (2003 winter, 164)				
Chongqing	0.14±0.08 (2003 winter, 198)	0.032±0.020 (2002-04, 14)	0.31±0.34 (2002-04, 14)	-	0.82±1.04 (2002-04, 14)
	0.40±0.17 (2003 summer, 202)				
Xi'an	0.12 (2006-7, 457)	0.009 (2006-07, 138)	0.010 (2006-07, 138)	-	0.013 (2006-07, 138)
Dalian	0.53 (2004, 136)	0.044±0.043 (2002-04, 89)	0.14±0.19 (2002-04, 89)	-	0.089±0.166 (2002-04, 89)
	0.19 (2004, 36)				
Hongkong	0.112±0.009 (2002, 100)	-	0.015±0.005 (2002, 100)	0.004±0.0003 (2002, 100)	0.003±0.0002 (2002, 100)
9 cities, including Beijing, Tianjin, Shanghai, Chongqing, Changchun, & Dalian	0.16±0.16 (2002-04, 1954)	0.12±0.27 (2002-04, 843)	0.26±0.67 (2002-04, 901)	0.11±0.19 (2002-04, 453)	0.19±0.56 (2002-04, 958)

[a] Adapted from Liu et al. 2012. [b] All values arithmetic means.

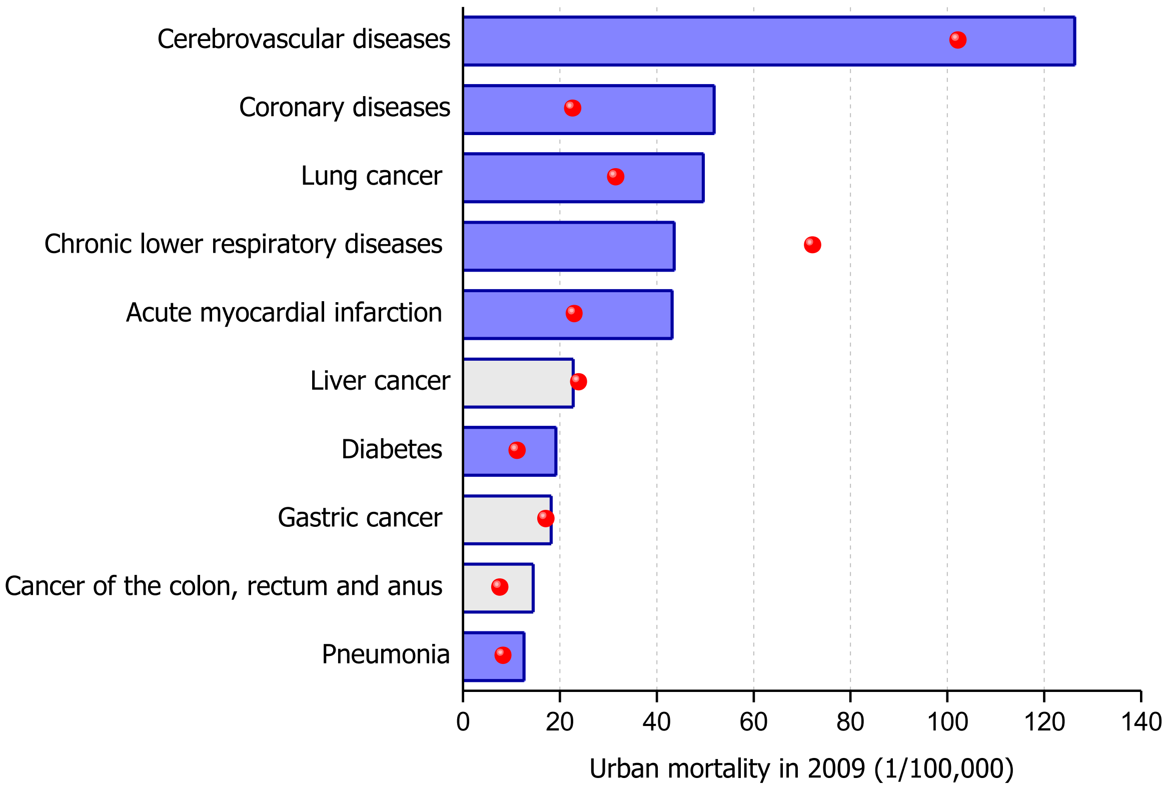

Supplemental Material, Figure S1. Top ten diseases responsible for mortality in urban China (Ministry of Health 2010). The solid blue bars indicate diseases where air pollution plays a role, contributing to the disease's development and/or progression. The red dots indicate urban mortality data for 2003.

References

Chen L, Mai B, Xu Z, et al. 2008. In- and outdoor sources of polybrominated diphenyl ethers and their human inhalation exposure in Guangzhou, China. Atmos Environ 42:78–86.

Guo Y, Kannan K. 2011. Comparative assessment of human exposure to phthalate esters from house dust in China and the United States. Environ Sci Technol 45(8): 3788-3794.

International Agency for Research on Cancer (IARC) 2012. A Review of Human Carcinogens: Chemical Agents and Related Occupations, IARC Monograph 100F, Benzene: pp 249-294; Formaldehyde: pp 401-435, World Health Organization, Lyon, France.

Lin XT, Shen T, Yu XL, Wang XY. 2009. Characteristics of phthalate esters pollution in indoor settled dust [In Chinese], J Environ Health 26: 1109-1111.

Liu ZR, Zhang JP, Li TT, Fang ZH, Zhou ZP, Bai YH. 2012. Investigation of indoor air pollutant concentrations in China [In Chinese]. In: Research advance report of indoor environment and health in China (Zhang YP, Deng QH, Qian H, Mo JH, eds). China Architecture & Building Press, Chapter 3,pp 37-46.

Lu XM, Zhang Q., Zhang XL, Dong HF, Zhang ZD, Wang BL. 2012. Determination of phthalate esters in house dust by gas chromatography/mass spectrometry [In Chinese]. Acta Univ Med Nanjing 32:1038-1042.

Ministry of Health – People's Republic of China. 2010. China Health Statistical Yearbook (2003-2009) [In Chinese].

Salthammer T. 2013. Formaldehyde in the ambient atmosphere: from an indoor pollutant to an outdoor pollutant? Angewandte Chemie 52, 3320-3327.

Wang B, Lee SC, Ho KF. 2007. Characteristics of carbonyls: Concentrations and source strengths for indoor and outdoor residential microenvironments in China. Atmos Environ 41(13): 2851-2861.

Wang FM, Chen L, Jiao J, Zhang LB, Ji YQ, Bai ZP, Zhang LW, Sun ZR. 2012. Pollution characteristics of phthalate esters derived from household dust and exposure assessment [in Chinese]. China Environmental Science 32(5): 780-786.

Weschler CJ, Nazaroff WW. 2008. Semivolatile organic compounds in indoor environments. Atmos Environ 42(40): 9018-9040.

Weschler CJ, Nazaroff WW. 2010. SVOC partitioning between the gas phase and settled dust indoors. Atmos Environ 44(30): 3609-3620.

Zhang Q, Lu XM, Zhang XL, Sun YG, Zhu DM, Wang BL, et al. 2013. Levels of phthalate esters in settled house dust from urban dwellings with young children in Nanjing, China. Atmos Environ 69: 258-264.
